# P-1593. Experience with a Rapid Diagnostic Blood Culture Identification Panel in Patients with Neutropenic Fever

**DOI:** 10.1093/ofid/ofae631.1760

**Published:** 2025-01-29

**Authors:** Dhruv Patel, Armaghan-E Rehman Mansoor

**Affiliations:** University of Kentucky, Ontario, California; University of Kentucky, Ontario, California

## Abstract

**Background:**

Rapid identification of organisms in bloodstream infections can expedite selection of appropriate antimicrobials. This can significantly impact patient care, especially in immunocompromised hosts. Our retrospective study evaluates a single-center experience with a rapid diagnostic blood culture panel (BCID) in patients with neutropenic fever.

**Figure:**
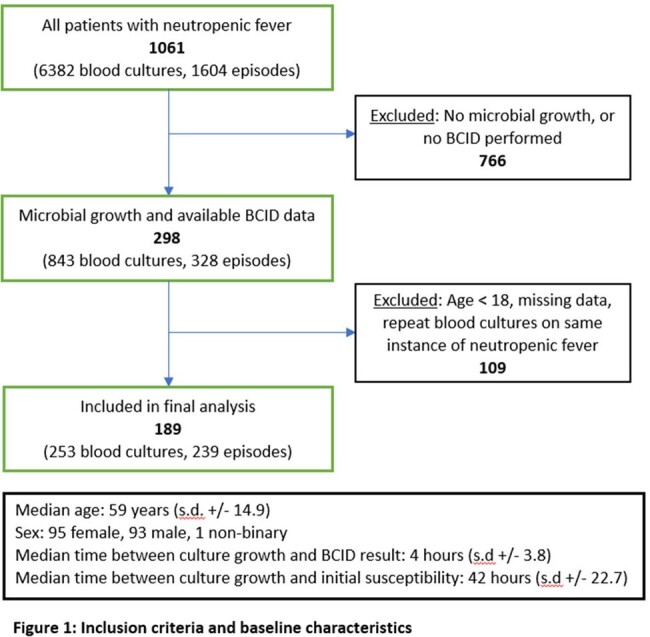

**Methods:**

Between January 2020 and October 2023, all adult patients with neutropenic fever for whom blood cultures were obtained were reviewed. As standard protocol, a reflex BCID was performed with the FilmArray© panel for all blood cultures with microbial growth. We evaluated concordance between cultures and BCID data, and clinical implication of discrepant results. Patient demographics, cause of neutropenia, and 90-day mortality was documented.

**Figure:**
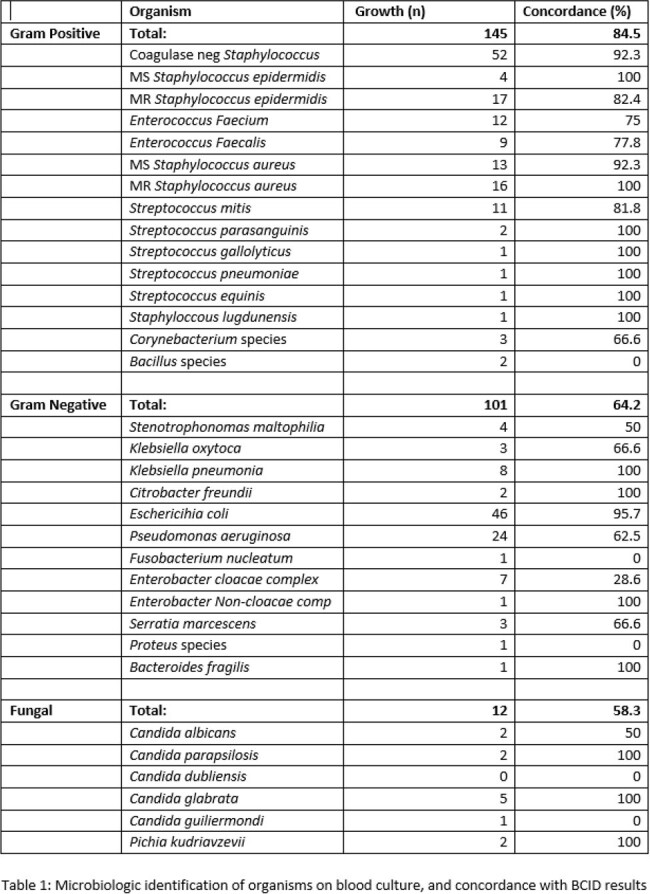

**Results:**

Of 6382 blood cultures in 1604 episodes for neutropenic fever, 253 unique blood cultures in 189 patients met criteria (Figure 1). Median age was 59 (SD±14.9) years, with 95 (50.3%) being female. A central venous catheter (CVC) was present in 75 patients (39.7%). The most common etiology for neutropenic fever was malignancy or chemotherapy in 124/189 (65.6%), followed by shock in 10/189 (5.3%).

BCID identified resistance genes in 86/253 (34.0%) of samples, with mecA (64, 25.3%) and CTX-M (11, 4.3%) most commonly reported. BCID and routine susceptibility testing showed similar results in 219/253 (86.6%) specimens (Table 1). Of the discordant results, 9/31 BCID results missed presence of an on-target multi-drug resistant organism (Table 2).

A change in therapy occurred based on BCID results from 143 (56.5%) of cultures in 94 (49.7%) of patients. There was no significant difference in 90-day mortality among patients where BCID led to an antibiotic change (37.2% vs 36.8%, p-value=0.96), and no difference in 90-day mortality among patients with discordant data (28.9% vs 37.5%, p-value=0.26).

**Figure:**
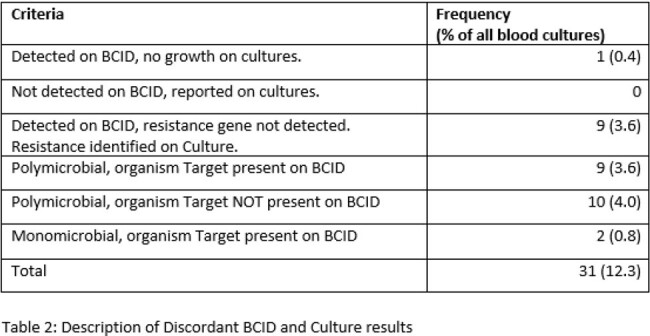

**Conclusion:**

In 239 episodes of neutropenic fever, BCID results correlated with routine results in 87% of blood cultures. BCID results resulted in quicker change to culture-appropriate antimicrobials in nearly half of the patients, however this was not associated with a mortality benefit.

**Disclosures:**

**All Authors**: No reported disclosures

